# Oncogenic microRNA-4534 regulates PTEN pathway in prostate cancer

**DOI:** 10.18632/oncotarget.12031

**Published:** 2016-09-15

**Authors:** Hannah Nip, Altaf A. Dar, Sharanjot Saini, Melissa Colden, Shahryari Varahram, Harshika Chowdhary, Soichiro Yamamura, Yozo Mitsui, Yuichiro Tanaka, Taku Kato, Yutaka Hashimoto, Marisa Shiina, Priyanka Kulkarni, Pritha Dasgupta, Mitsuho Imai-Sumida, Z. Laura Tabatabai, Kirsten Greene, Guoren Deng, Rajvir Dahiya, Shahana Majid

**Affiliations:** ^1^ Department of Urology, VA Medical Center and UCSF, San Francisco, California, USA; ^2^ Research Institute, California Pacific Medical Center, San Francisco, California, USA

**Keywords:** microRNA, prostate cancer, oncogene, PTEN, miR-4534

## Abstract

Prostate carcinogenesis involves alterations in several signaling pathways, the most prominent being the PI3K/AKT pathway. This pathway is constitutively active and drives prostate cancer (PCa) progression to advanced metastatic disease. *PTEN*, a critical tumor and metastasis suppressor gene negatively regulates cell survival, proliferation, migration and angiogenesis via the PI3K/Akt pathway. PTEN is mutated, downregulated/dysfunctional in many cancers and its dysregulation correlates with poor prognosis in PCa. Here, we demonstrate that microRNA-4534 (miR-4534) is overexpressed in PCa and show that miR-4534 is hypermethylated in normal tissues and cell lines compared to PCa tissues/cells. miR-4534 exerts its oncogenic effects partly by downregulating the tumor suppressor PTEN gene. Knockdown of miR-4534 impaired cell proliferation, migration/invasion and induced G0/G1 cell cycle arrest and apoptosis in PCa. Suppression of miR-4534 and its effects on tumor growth was confirmed in a xenograft mouse model. We performed parallel experiments in non-cancer RWPE1 cells by overexpessing miR-4534 followed by functional assays. Overexpression of miR-4534 induced pro-cancerous characteristics in this non-cancer cell line. Statistical analyses revealed that miR-4534 has potential to independently distinguish malignant from normal tissues and positively correlated with poor overall and PSA recurrence free survival. Taken together, our results show that depletion of miR-4534 in PCa induces a tumor suppressor phenotype partly through induction of PTEN. These results have important implications for identifying and defining the role of new PTEN regulators such as microRNAs in prostate tumorigenesis. Understanding aberrantly overexpressed miR-4534 and its downregulation of PTEN will provide mechanistic insight and therapeutic targets for PCa therapy.

## INTRODUCTION

Prostate cancer is the second most prevalent cause of cancer deaths among males in the United States [1] and the fourth most common tumor type worldwide [2]. According to recent data, it is estimated that 220, 800 newly diagnosed prostate cancer cases and 27, 540 deaths will occur in 2015 [1]. The five year relative survival rate of early stage prostate cancer is > 99% while that of advanced metastatic disease is only 28% [1]. The clinical behavior of localized prostate cancer is highly variable, while some men have aggressive cancer leading to metastasis and death from the disease, many others have indolent cancers that are cured with initial therapy or may be safely observed. Multiple risk stratification systems have been developed, combining the best currently available clinical and pathological parameters (such as Gleason score, PSA levels and clinical and pathological staging); however, several areas of urgent unmet need remain [3–5], e.g., a validated biomarker to complement PSA for screening, prognostic biomarkers with clinical utility, further risk stratification using molecular differentiation could potentially help distinguish indolent from aggressive prostate cancer. Prostate carcinogenesis features alterations in several signaling pathways, the most prominent of which is the highly oncogenic prosurvival PI3K/AKT signaling pathway [6]. In human prostate cancer, the PI3K/Akt pathway is frequently activated due to inactivation of *PTEN*.

*PTEN* (phosphatase and tensin homolog deleted on chromosome 10; also known as *MMAC1* and *TEP1*) is one of the most frequently altered tumor suppressor genes in cancer, and in particular, prostate cancer [7, 8]. It catalyzes the conversion of the membrane lipid second messenger PIP3 to PIP2 and is therefore a key mediator of the PI3K/Akt pathway [9]. PTEN levels have been identified as being critical in determining the mechanism by which prostate tumors evolve *in vivo* [10, 11]. PTEN heterozygosity has been shown to promote tumor initiation and proliferation [12]. In addition, primary tumors often show loss or alteration of at least one PTEN allele in 70%–80% of primary prostate cancers [2, 13], while homozygous inactivation of PTEN is generally associated with advanced cancer and metastasis [14, 15]. Functionally, PTEN is a nonredundant, plasma-membrane lipid phosphatase and its inactivation upregulates PI3K/AKT signaling enhancing protein synthesis, cell migration and tumor-induced angiogenesis [16–22]. PTEN has been reported to be repressed by oncogenic microRNAs (miRNA), e.g., miRNA-21, −214, −301 in many cancers [23–26], suggesting that oncogenic activity of these miRNAs is in part through downregulation of PTEN. Although not much work has been done on oncogenic miRNAs as compared to tumor suppressor miRNAs, it is very interesting that miRNAs may influence carcinogenesis through PTEN regulation.

MicroRNAs (miRNAs) are a naturally-occurring class of short, non-coding RNA molecules between 19 and 21 nucleotides long [27]. In humans approximately 2,8645 unique mature miRNAs have been identified (http://mirdb.org/miRDB/). While the exact function of miRNAs remain to be fully elucidated, they are known to regulate gene expression via binding target messenger RNA (mRNA), inhibiting translation or triggering mRNA degradation. Importantly, it has been demonstrated that in addition to their inhibitory role, miRNAs can also induce or activate transcript levels [28–30]. Through these mechanisms miRNAs perform a regulatory role in various cellular processes, including cell development, differentiation, proliferation and apoptosis [29, 31, 32].

Dysregulated miRNA expression patterns have been noted in many organisms, encompassing a wide spectrum of pathological processes, from immunological defects in fish, altered developmental phase transition and flowering time in plants, and neurodegeneration, cardiovascular disease and cancer in humans [33, 34]. Discovery of dysregulated miRNA expression has led to the hypothesis that miRNAs could potentially be used as disease diagnostic or prognostic markers. Furthermore, miRNA are attractive therapeutic targets for the treatment of various conditions, including cancer. At present, multiple clinical trials are currently registered with ClinicalTrials. gov, investigating the ability of miRNA to function as disease biomarkers and response to current therapies. The novel role of miRNA and their importance in many different processes has led to an explosion of scientific enquiry into miRNA function. Thus the aim of this study is to understand the role of microRNA-4534 (miR-4534) dysregulation in promoting prostate oncogenesis. miR-4534 was selected based on our previous study [35] that we performed to preliminary screen and identify differentially expressed miRNAs in prostate cancer cell lines compared to a non-malignant cell line. A set of miRNAs, miR-205, −203, 23b and −34b were found to be significantly downregulated whereas miR-4534 was significantly upregulated in prostate cancer cells compared to a non-malignant cell line.

In this study we report that: (i) miR-4534 is overexpressed in prostate cancer tissues and cell lines compared to matched normal samples and cell lines; (ii) miR-4534 has potential to distinguish malignant from normal tissues; (iii) miR-4534 functions as an oncomiR as its attenuation results in cell cycle arrest, apoptosis, impaired migration/invasion and colony forming capability of prostate cancer cells; (iv) miR-4534 directly targets PTEN and its downstream effectors to exert its functional effects; (v) intra-tumoral delivery of anti-miR-4534 (anti4534) inhibits *in-vivo* tumor growth in nude mice xenografts.

## RESULTS

### miR-4534 upregulation is correlated with methylation status in prostate cancer

**Figure 1:**
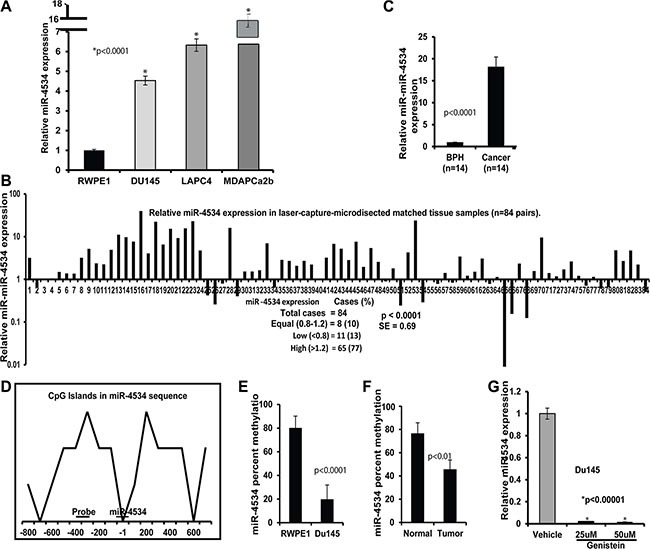
miR-4534 expression and methylation status in prostate cancer (**A**) Quantitative RT-PCR analysis of miR-4534 in cell lines. (**B**–**C**) Quantitative real time PCR analysis of mir-4534 expression in 84 pairs of matched Laser-Captured Microdissected tissue samples (B) and non-matched BPH and cancer cohort (C). (**D**) CpG island within the 1.0 kb region upstream of miR-4534 gene. Sequences of methylation primers and probes are given in Supplementary Table S2. (**E**–**F**) Mir-4534 methylation percentage in Du145 prostate cancer cell line compared to non-cancer RPWEI cells (E) and matched tissue samples (F). (**G**) Negative regulation of mir-4534 expression by genistein treatment in Du145 cell line.

In a previous study [35], we performed preliminary screening to identify differentially expressed miRNAs in prostate cancer cell lines compared to a non-malignant cell line. A set of miRNAs, miR-205, −203, 23b and −34b were found to be significantly downregulated whereas miR-4534 was significantly upregulated in prostate cancer cells compared to a non-malignant cell line. We validated this data by miRNA-quantitative real time PCR (miR qRT-PCR) analysis. The results confirmed that miR-4534 was overexpressed in prostate cancer cell lines compared to normal RWPE1 cells (Figure 1A). Further, miR-4534 expression was analyzed in 168 (84 pairs) of laser-captured micro-dissected (LCM) matched clinical samples (Figure 1B), an unmatched group of 14 benign prostatic hyperplasia (BPH) and 14 tumor samples (Figure 1C). Among all tissues miR-4534 expression was significantly upregulated in cancer samples compared to normal or BPH tissues (Figure 1A–1C). These results indicate a putative oncogenic role for miR-4534 in prostate cancer.

Bert et al. [36] published an interesting study in “Cancer Cell” in which they report that extensive DNA hypermethylation of CpG islands is strongly related to cancer-specific gene activation. Guided by this study, we were curious to know the methylation status of miR-4534 in normal and prostate cancer and identified a CpG island in the upstream 1kb sequence of miR-4534 (Figure 1D). Interestingly, we observed hypermethylation of miR-4534 in a normal cell line compared to the Du145 cancer cell line (Figure 1E). We also examined the methylation status in several tissue samples and the results were consistent with hypermethylation observed in normal samples compared to their matched cancers (Figure 1F). Since our group has been working on the effect of genistein-a natural chemotherapeutic agent on various cancer cells, we also looked at the effect of genistein on prostate cancer cells and observed that genistein treatment of Du145 cancer cells suppressed miR-4534 expression levels (Figure 1G). Thus the methylation status is in agreement with the expression profile of miR-4534 in normal and prostate cancer cells and tissues.

### Clinical significance of miR-4534 in prostate cancer

**Figure 2:**
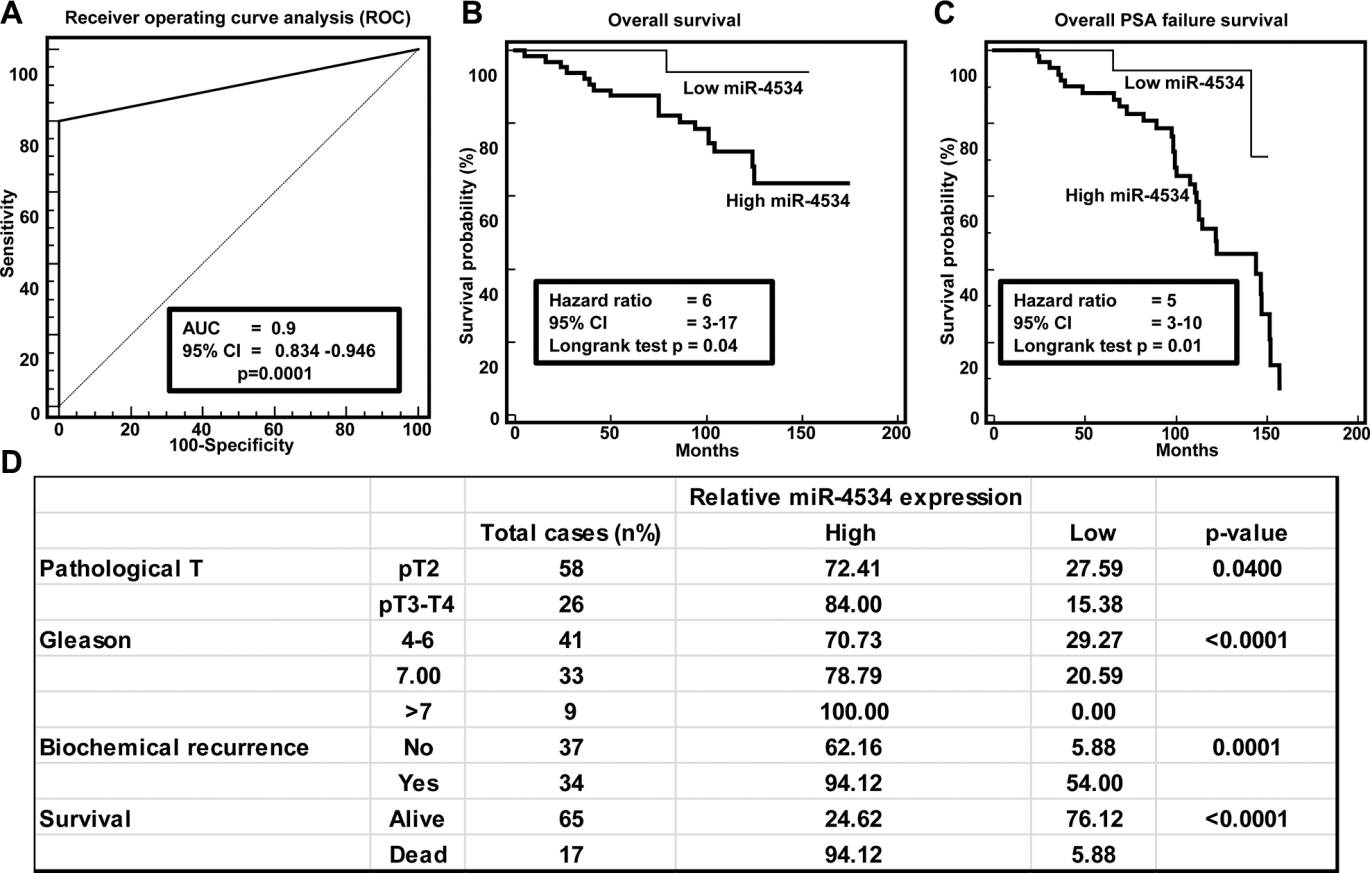
Clinical significance of miR-4534 in prostate cancer (**A**) ROC curve analysis showing performance of miR-4534 expression to discriminate between malignant and non-malignant tissue samples. (**B**–**C**) Kaplan-Meier analysis for overall survival and recurrence free survival based on miR-4534 expression. (**D**) Chi-square test showing correlation of clinicopathological characteristics with miR-4534 expression.

Clinical demographics of the study cohort are summarized in Supplementary Table S1. Receiver operating curve (ROC) analyses were performed to evaluate the ability of miR-4534 expression to discriminate between normal and tumor cases. An area under the ROC curve (AUC) of 0.9 (*P <*0.0001; 95% CI = 0.834 to 0.946) (Figure 2A) was obtained suggesting that miR-4534 expression can discriminate between malignant and non-malignant samples and hence has potential to be used as a diagnostic marker for PCa, though it needs to be validated in a larger independent cohort. To determine whether miR-4534 has prognostic significance, we divided 84 cases into low miR-4534 (expression T/N < 0.8 fold) and high miR-4534 (expression T/N > 0.8 fold) groups and performed Kaplan-Meier survival analysis. The results show that the low miR-4534 group displayed significantly higher overall survival probability compared to the high miR-4534 group (Logrank Test *p <* 0.04, HR = 6, 95% CI = 3–17) (Figure 2B). Kaplan-Meier survival analysis for recurrence free survival was performed for the same sample cohort and cases with low miR-4534 expression had better recurrence free survival than with high miR-4534 expression cases (Logrank Test *p <* 0.01, HR = 5, 95% CI = 3–10) (Figure 2C). We also determined the correlation of miR-4534 expression with clinicopathological variables such as Gleason grade, pathological stage (pT), biochemical recurrence and survival (Figure 2D). Correlation tests revealed that cases with high miR-4534 expression increased from low grade, low pathological stage to high grade and high pathological stage (Figure 2D). Patients that had PSA recurrence also had significantly high miR-4534 expression.

### Attenuation of miR-4534 impairs cell proliferation and induces cell cycle arrest and apoptosis in prostate cancer

**Figure 3:**
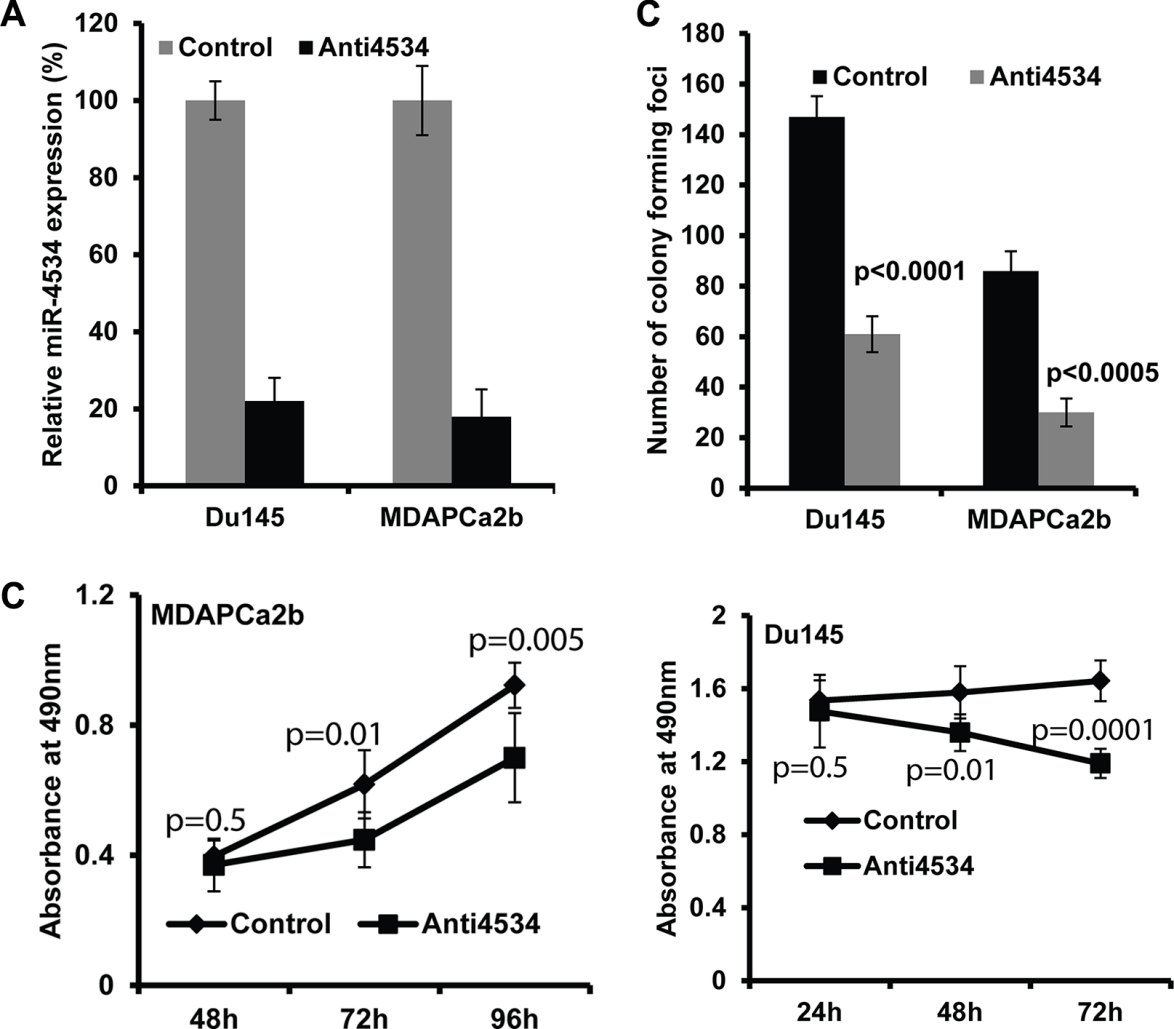
Effect of miR-4534 knockdown in prostate cancer cells (**A**) Expression of miR-4534 post transient transfection of 50 nM miR-4534 inhibitor (anti4534) in prostate cancer cells compared to anti-miR-control inhibitor (control). (**B**) Proliferation of MDAPCa2b and Du145 cells. After anti4534 transfection cell proliferation was significantly reduced compared to control. (**C**) miR-4534 depletion significantly inhibits prostate cancer cell colony formation.

**Figure 4:**
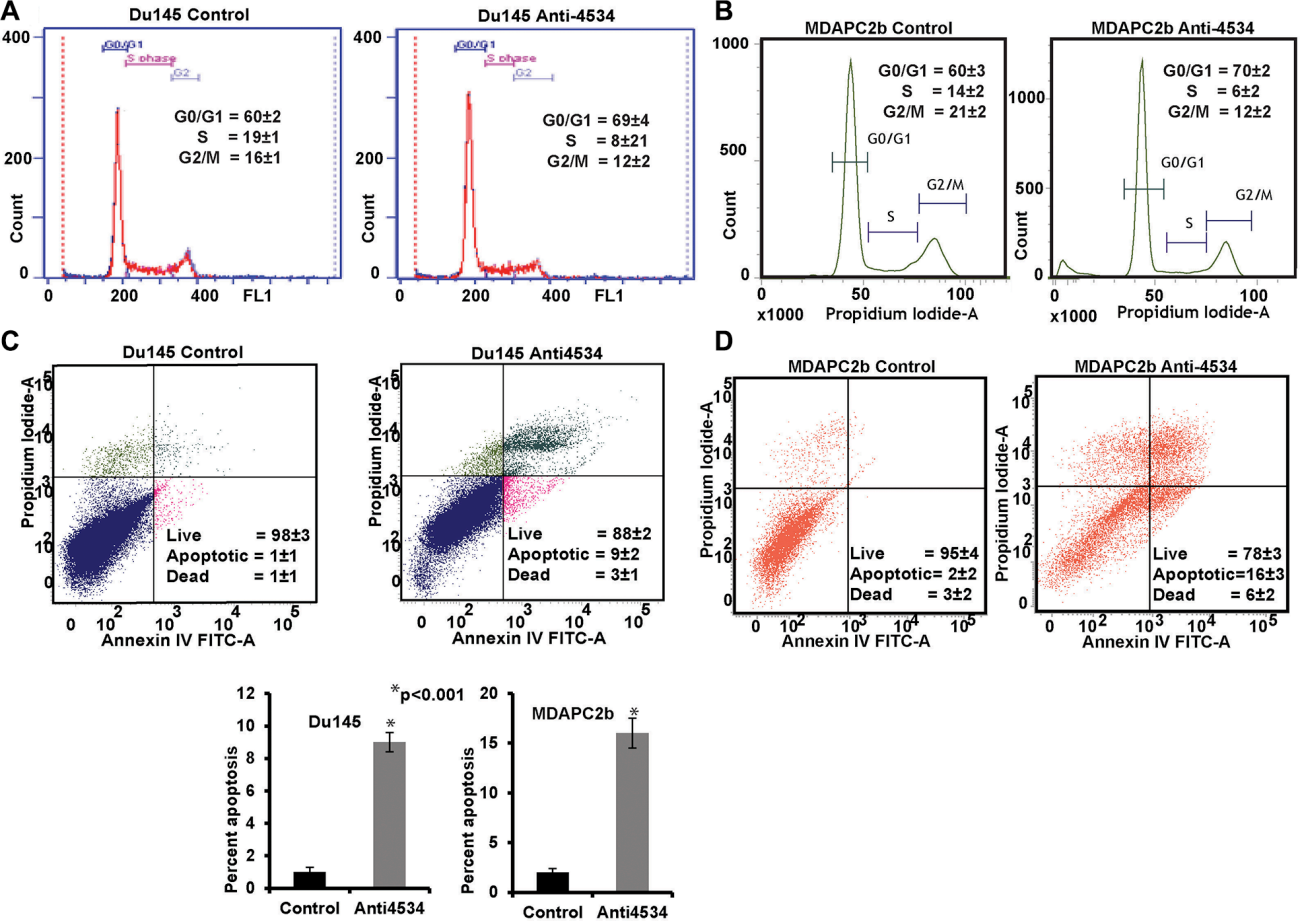
Knockdown of miR-4534 induces G0/G1 cell cycle arrest and apoptosis in prostate cancer cells (**A**–**B**) Cell cycle analysis showing an increase in the G0/G1 phase of Du145 and MDAPCa2b cells after transfection with anti4534. (**C**–**E**) Apoptosis assay showing induction of apoptosis by anti4534. *p < 0.05, ± CI.

To determine the functional significance of miR-4534 in prostate cancer, we transfected prostate cancer cell lines with miR-4534 inhibitor (anti4534; 50 nM) (Figure 3A) or a control inhibitor for 72 or 96 hours as indicated followed by functional assays. Transient transfection of cells with anti4534 resulted in significantly decreased cell proliferation over time (Figure 3B) as compared to cells expressing anti-control-miR sequence (control). We further examined the effects of anti4534 on prostate cancer cell viability using a colony formation assay. The anti4534-transfected cells showed low colony formation ability, as both the size and number of foci in anti4534 expressing cells was suppressed when compared to control expressing cells (Figure 3C). Cell cycle analysis revealed a significant increase in G0/G1 cell population (9–10%) with a concomitant decrease in the S-phase (8–11%) of prostate cancer cells transfected with anti4534 compared to control (Figure 4A–4B). Repression of miR-4534 also induced apoptosis in cancer cells as compared to control (8–14%) (Figure 4C–4E). These results confirm the phenotypic effects of miR-4534 downregulation in human prostate cancer cells.

### Knockdown of miR-4534 suppresses prostate cancer cell migration and invasion

**Figure 5:**
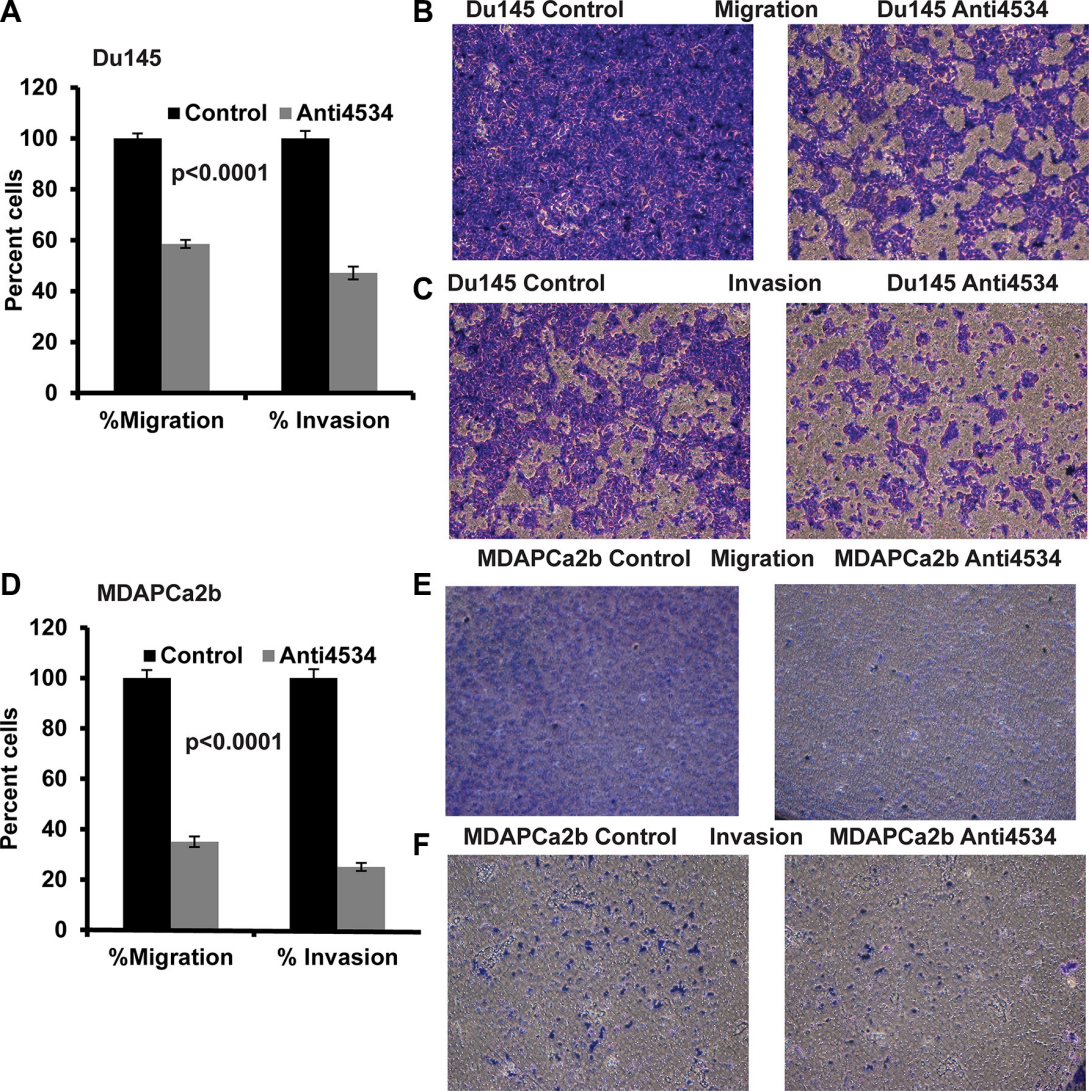
Knockdown of miR-4534 inhibits migration and invasion in prostate cancer cells (**A**–**C**) Migration and invasion assays in Du145 cells transfected with anti4534 compared to anti-negative control miR (control). (**D**–**F**) A significant decrease in the percent migrating or invading MDAPCa2b cells was observed with anti4534 compared to control.

Transient transfection of Du145 cells with anti4534 resulted in a significant decrease in migration (40%, *p <* 0.0001) and invasion (50%, *p <* 0.0001) post 72 h transfection (Figure 5A–5C) and the results were consistent with the MDAPCa2b cancer cell line with a decrease in migration (60%, *p <* 0.0001) and invasion (70%, *p <*0.0001) after 96 h transfection (Figure 5D–5F) compared to control cells. These findings suggest that loss of miR-4534 expression impairs migration and invasion of prostate cancer cell lines.

### miR-4534 directly represses the tumor suppressor PTEN gene and its down-stream effectors involved in proliferation/survival/invasion and migration pathways

**Figure 6:**
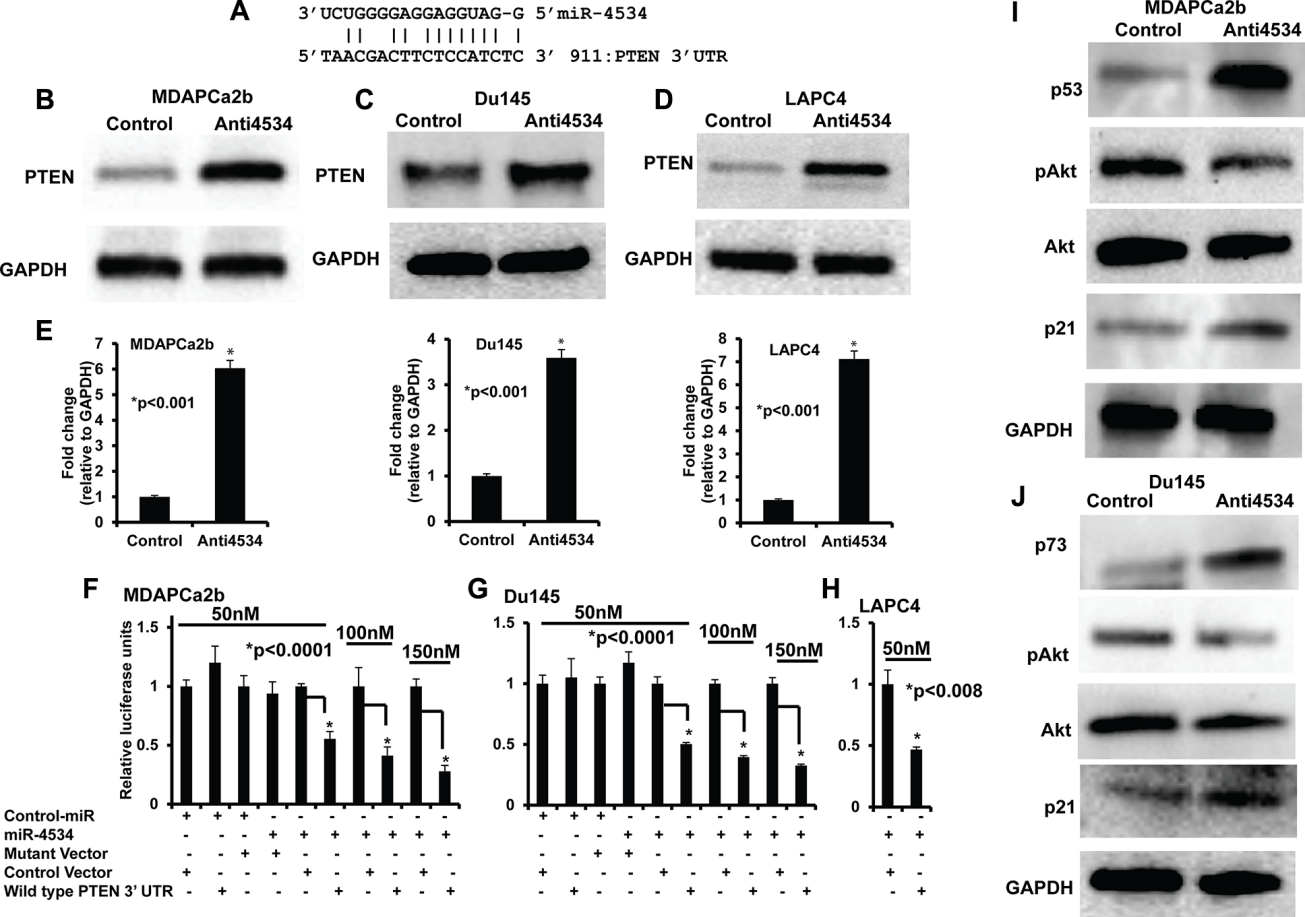
miR-4534 directly targets tumor suppressor PTEN and regulates downstream pathway genes involved in cell proliferation, survival and migration (**A**) Complimentary binding site for miR-4534 in 3′UTR of PTEN gene. (**B**–**E**) Transient transfection of anti4534 caused a significant increase in PTEN protein levels compared to control. (**F**–**H**) Luciferase assays showing decreased reporter activity after co-transfection of either wild type PTEN-3′UTR or its mutated 3′UTR with miR-4534 in prostate cancer cells. (**I**–**J**) Western blot analysis showing decreased expression of PTEN downstream genes involved in the survival pathway.

Based on the phenotypic effects of miR-4534 depletion, we focused on the putative gene targets of miR-4534 in prostate cancer. We utilized *in silico* computational algorithms (miRbase.org) and identified complimentary binding sites in the 3′UTR of PTEN (Figure 6A), a master negative regulator of the pro-survival PI3K/Akt pathway. Western analysis showed that downregulation of miR-4534 attenuated PTEN protein expression in three prostate cancer cell lines (Figure 6B–6D). To validate if miR-4534 directly targets PTEN, we performed 3′UTR luciferase reporter assays with the three cell lines (Figure 1F–1H). Co-transfection of the indicated concentrations of miR-4534 along with wild type 3′UTR of PTEN significantly repressed relative luciferase activity (Figure 1F–1H), whereas no effect was observed with cont-miR or miR-4534 transfected with a mutated PTEN 3′UTR, suggesting that miR-4534 directly targets the wild type 3′UTR of the PTEN tumor suppressor gene. Next we determined the effect of miR-4534 mediated suppression of PTEN on the downstream pathway genes. Results indicated that induction of PTEN by knockdown of miR-4534 resulted in the upregulation of p53, p73, and p21 protein that are involved in prostate cancer cell growth and progression. Depletion in posphoAkt levels was also observed, a critical molecule in tumor development, cell survival and proliferation (Figure 6I–6J). These results support our hypothesis that miR-4534 is an onco-miR that exerts its phenotypic effects partly through PTEN and the downstream PI3K/Akt pathway in prostate cancer.

### Overexpression of miR-4534 causes pro-cancerous properties in non-cancerous RWPE1 cells

**Figure 7:**
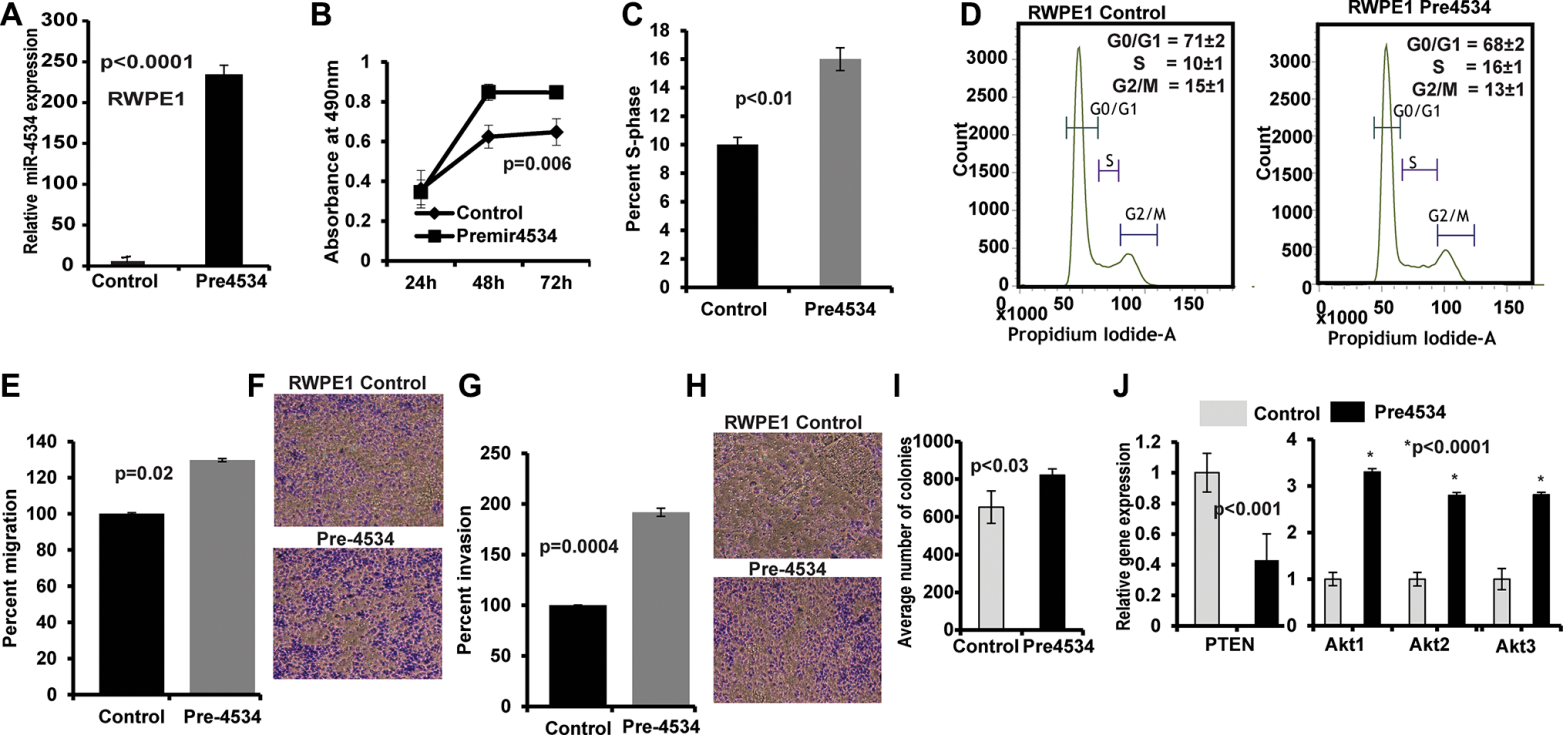
Overexpression of miR-4534 in normal prostate cells (RWPE1) (**A**) miR-4534 expression after transient transfection of pre-miR-4534 compared to pre-miR-negative control (control). (**B**) miR-4534 overexpression in normal prostate cells induced cell proliferation. (**C**–**I**) An increase in the s-phase cell population (C–D) along with increased migration (E–F), invasion (G–H) and colony formation (I) capability of RPWE1 cells after overexpression of miR-4534 mimic. (**J**) Relative mRNA expression of PTEN, Akt1-3 in miR-4534 overexpressing RWPE1 cells compared to control. GAPDH was used as an endogenous control.

To determine the biological significance of miR-4534 in prostate cancer, we performed parallel experiments in the non-cancerous RWPE1 cell line. miR-4534 was over-expressed in RWPE1 cells by transient transfection of miR-4534 mimic (premiR-4534; 50 nM) along with a negative-miRNA-control (control) (Figure 7A). RWPE1 cells transfected with premiR-4534 showed a considerable increase in proliferation (Figure 7B) compared to control. Moreover, there was an increase in the S-phase of the cell cycle including enhanced migration, invasion and colony formation capability of cells transfected with pre-miR-4534 compared to control (Figure 7C–7I). These data provide evidence that overexpression of miR-4534 caused RWPE1 cells to have cancer like characteristics. We further determined the effect of overexpression of miR-4534 on PTEN and Akt by qRT-PCR. As expected there was a decrease in relative expression of PTEN with an increase in Akt1-3 relative expression. GAPDH was used as an endogenous control.

### Intra-tumoral delivery of anti4534 suppresses tumorigenecity *in vivo*

**Figure 8:**
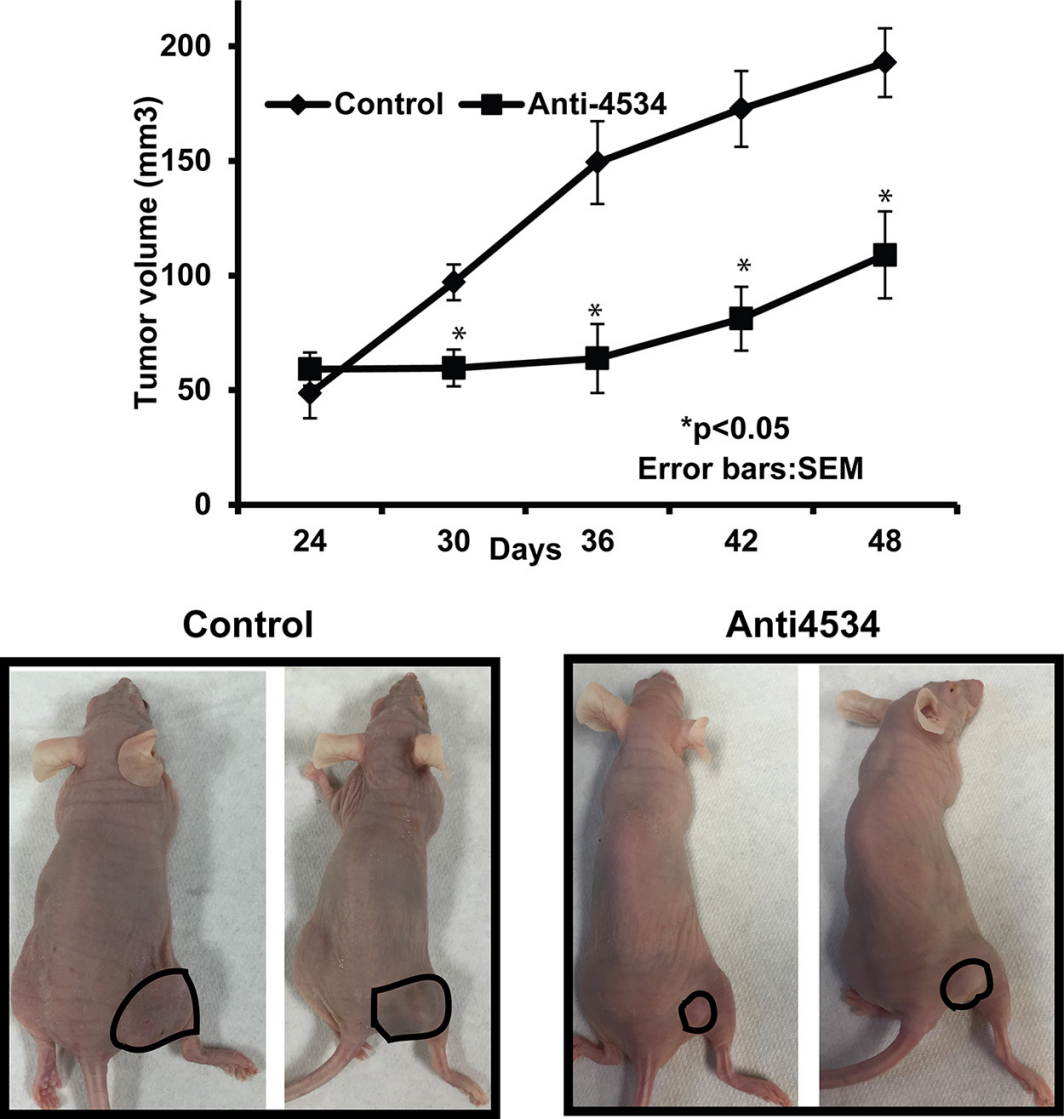
Inhibition of *in vivo* tumor growth by intratumoral injection of anti4534 *p < 0.05 (**A**) Tumor volume following intratumoral injection of control or anti4534 into established tumors. Data represents the mean of each group and error bars are S.E.M. (**B**) Representative pictures of mice injected with either control or miR-4534 at the termination of experiment.

We also performed *in-vivo* growth suppression experiments to determine the tumor suppressive effect of miR-4534 depletion by local administration of anti4534 or control in established tumors. Tumor growth was significantly suppressed by anti4534 over the course of the experiment compared to controls. Average tumor volume in controls was 132 mm^3^ compared to the average tumor volume of 74 mm^3^ in mice that received anti4534 (Figure 8) at the termination of the experiment. These results confirm the *in vitro* tumor suppressive effect of downregulation of miR-4534 in a prostate xenograft model.

## DISCUSSION

miR-4534 is located at Chromosome22q13.1 (Chr22q13.1). The loci Chr22q13 and Chr22q12.3-q13.1 have been reported to be associated with prostate cancer aggressiveness [37, 38]. Very little is known about miR-4534 and a pub-Med search resulted in one publication that was performed in Type 2 diabetes [39]. We also utilized online publicly available data bases to look for any information about miR-4534. We found that miR-4534 was shown to be amplified in 2% of tumor samples in the Prostate Adenocarcinoma (Nelson Lab, Fred Hutchinson CRC) sample cohort (http://www.cbioportal.org/). While the online data base “The European Bioinfomatics Institute” (EBI; http://www.ebi.ac.uk/) shows that the expression of miR-4534 is less in normal prostate. In our preliminary screening analysis, we found miR-4534 to be upregulated in cancer cells compared to normal RWPE1 cells. We confirmed overexpression of miR-4534 in laser-captured micro-dissected prostate cancer clinical samples and cancer cell lines compared to matched normal tissues and a non-cancerous cell line (RWPE1) by quantitative-real-time-PCR (qRT-PCR). Thus our data is consistent with the EBI report that miR-4534 is low in normal prostate tissues compared to matched cancer tissues. However, to the best of our knowledge no study has been performed on the status and functional role of this novel miRNA in cancer.

Our results reveal that miR-4534 expression has clinical significance in prostate cancer as it can independently differentiate malignant from normal tissues. Higher miR-4534 expression positively correlated with poor overall and recurrence free survival. From a clinical point of view, miRNAs possess several features that make them attractive candidates as new diagnostic and prognostic biomarkers of cancer [40] and miRNA signatures have been reported to be useful tools for early diagnosis of cancer [41, 42]. In this study, we found that miR-4534 has potential to be a diagnostic biomarker for prostate cancer and for predicting biochemical relapse (*p <* 0.01) in patients. These results need to be confirmed in a larger independent cohort of patient samples.

**Figure 9:**
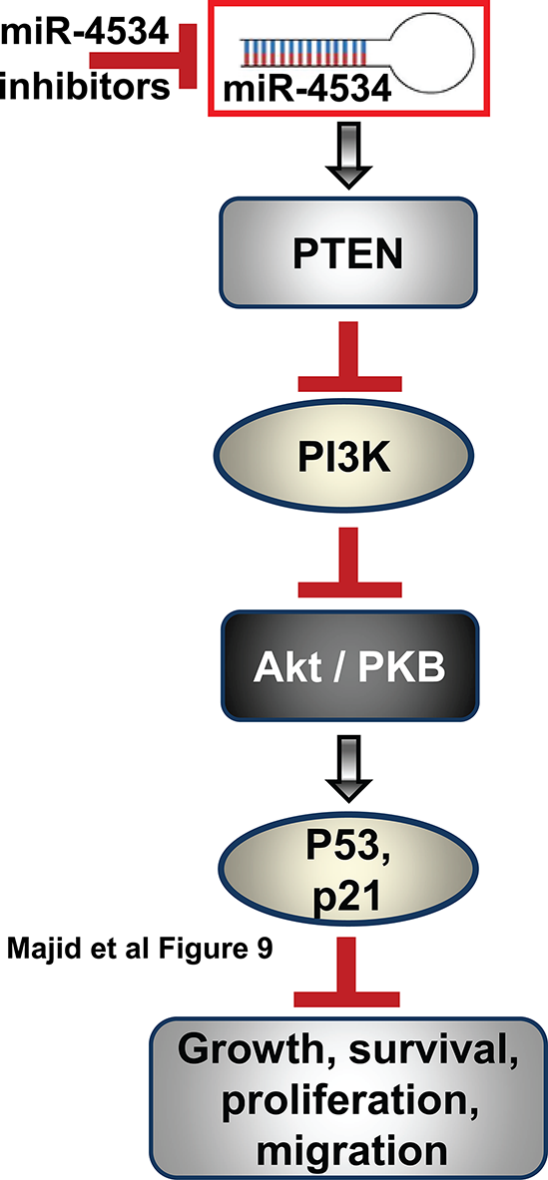
Schematic representation of role of miR-4534 in prostate cancer Inhibition of miR-4534 leads to induction of PTEN that leads to inhibition of PI3K/Akt pathway causing inhibition of growth, survival, proliferation and migration of prostate cancer cells.

We found that miR-4534 impaired cell viability and proliferation of prostate cancer cells and G0/G1 cell cycle arrest was observed with the induction of apoptosis. In addition, a decrease in cell migration and invasion was noted after transient transfection of anti4534 in prostate cancer cells. These results confirm the oncogenic role of miR-4534 as its depletion caused tumor suppression in prostate cancer cells. miRNAs being non-coding RNAs exert their functional effects by regulating gene expression via binding target messenger RNA (mRNA) and inhibiting its translation or triggering mRNA degradation [28, 31, 32]. We utilized in-silico algorithms to find miR-4534 target genes in the PI3K/Akt survival pathway and found that PTEN has miR-4534 binding sequence in its 3′UTR. Immunoblot assay showed significant upregulation of PTEN protein after repression of miR-4534 in prostate cancer cells. Luciferase reporter assays with three prostate cancer cell lines confirmed that miR-4534 directly targets the PTEN gene. Hence this study shows that miR-4534 exerts its oncogenic effects partly by downregulating the tumor suppressor PTEN gene. It is possible that miR-4534 might also target additional target genes since a single miRNA can target a broad range of molecular regulators in a context-dependent manner. Functional loss of PTEN leads to aberrant activation of the oncogenic PI3K/Akt pathway and is instrumental in promoting cancer development, progression and metastasis [16, 43–48]. Thus, restoration of PTEN function represents a major therapeutic strategy for the management of prostate cancer (Figure 9). We found that depletion of miR-4534 caused an induction in PTEN expression that resulted in G0/G1cell cycle arrest and increased apoptosis with inhibition of migration and invasion in prostate cancer cells. In addition, anti4534 mediated induction of PTEN decreased downstream pAkt and induced p21, p53 and p73 that are involved in cell survival, apoptosis, proliferation, growth, migration and angiogenesis [16, 17, 49–58]. This study reveals that miR-4534 promotes the oncogenic PI3K/Akt module by inhibiting the PTEN gene in prostate cancer. The PTEN/PI3K/Akt pathway is amenable to pharmacological manipulation, and PTEN itself may become a drug target as is the case for p53 [16, 59, 60]. The discovery of new regulators, such as miRNAs that influence PTEN expression and a greater understanding of their regulatory networks will assist in employing the bonafide tumor suppressor PTEN gene to inhibit prostate tumorigenesis.

We also examined the biological significance of miR-4534 in a non-cancerous prostate cell line (RWPE1) and found that overexpression of miR-4535 induces pro-cancerous characteristics highlighting the importance of this miRNA in the oncogenesis of prostate cancer. The anti-tumorigenic effects of depletion of miR-4534 observed in this study were also confirmed in a PCa xenograft nude mouse model. *In vivo* experiments demonstrated a striking suppression in subcutaneous tumor growth in nude mice where anti4534 was directly administered to tumors. Therefore, our study demonstrates that targeting this novel oncogenic miR-4534 inhibits prostate cancer growth and progression both *in vitro* and *in vivo*.

In summary, our study has identified novel oncogenic miR-4534 harbored at locus Chr22q13.1 that is implicated in aggressive prostate cancer. miR-4534 expression has clinical significance to be a diagnostic/prognostic biomarker as it can diagnose prostate cancer from normal tissues and is positively correlated with poor overall and recurrence free survival. We also found that miR-4534 is overexpressed in prostate cancer and directly targets PTEN leading to its downregulation. Furthermore, induction of PTEN could be achieved by miR-4534 depletion, which caused significant inhibition of tumorigenesis in prostate cancer cells. We conclude that therapeutic modulation of miR-4534 in prostate tumors provides an alternative option for the management of prostate cancer.

## MATERIALS AND METHODS

### Cell culture, plasmids and probes/primers

Human prostate cancer cell lines MDAPCa2b, Du145 and a non-malignant prostate cell line RWPE1 were obtained from the American Type Culture Collection (ATCC) (Manassas, VA) and grown according to ATCC protocol. These human-derived cell lines were authenticated by DNA short-tandem repeat analysis by the ATCC. TaqMan probes for hsa-miR-4534, negative control pre-miR, inhibitor of miR-4534 and anti-control inhibitor (control) were purchased from Applied Biosystems (Foster City, CA). For luciferase reporter assays, pMIR-REPORT luciferase vector was purchased from Ambion, Cambridge MA. Genistein was purchased from Sigma (Sigma, St. Louis, MO) and dissolved in DMSO. Genistein treatment was performed as described previously [61]. Briefly, subconfluent cells (60–70% confluent) were treated with varying concentrations of 25 and 50 umol/L genistein and cells treated with vehicle (DMSO) served as control. The cells were treated with fresh genistein along with change of media and grown for 3 days.

### Quantitative real-time PCR

Tissue samples from radical prostectomy were obtained from the Veterans Affairs Medical Center, San Francisco, CA, USA. Total RNA was extracted and assayed for mature miRNAs and mRNAs using the TaqMan MicroRNA Assays and Gene Expression Assays, respectively, in accordance with the manufacturer's instructions (Applied Biosystems). All RT reactions were run in a 7500 Fast Real Time PCR System (Applied Biosystems). Relative expression was calculated using the comparative Ct.

### Methylation analysis of miR-4534 by quantitative methylation-specific PCR (qMSP)

To investigate the methylation status of miR-4534 in PCa we performed methylation analysis in in cell lines and tissue samples. DNA was bisulfite converted using EZ DNA Methylation-Gold Kit (Zymo Research, Orange, CA, USA) according to the manufacturer's protocol. The converted DNA was amplified by PCR with 400 pM of primer set F1/R1, and HotStar Taq Plus DNA Polymerase (Qiagen, Valencia, CA, USA). PCR was performed by denaturation at 95^°^C for 5 minutes, followed by 15 cycles of 94^°^C for 30 seconds, 56^°^C for 30 seconds and 72^°^C for 30 seconds. 2 μl of the PCR product was added to 40 μl solution containing 20 μl TaqMan Fast Universal PCR Master Mix (2×) (Applied Biosystems), 500 pM primers F1/R1. The mixed solution was aliquoted evenly into two tubes, and was added 1 μl 5 μM probe for methylation reaction (PM) for Methylation (M) reaction and 1 μl 5 μM probe for unmethylation reaction (PU) probe for Unmethylation (U) reaction, respectively. Methylation was measured by realtime quantitative PCR with an Applied Biosystems 7500 Fast Sequence Detection. For each sample, the percent of methylation was calculated by the difference of Ct in M reaction (Ct-M) and Ct in U reaction (Ct-U). The probes for methylation/unmethylation specific realtime PCR were synthesized by Applied Biosystems (Foster City, CA, USA), labeled with 6FAM reporter at 5′ end and with MGB quencher at 3′ end. The sequences of primers and probes are given in Supplementary Table S2.

### Flow cytometry, cell viability, migration, clonability and invasion assays

FACS analysis for cell cycle and apoptosis was done 72 hours (96 hrs for MDAPCab cells) post-transfection using nuclear stain DAPI for cell cycle analysis or ANNEXIN V-FITC /PI KIT (Betkin Dikeson) for apoptosis analysis according to the manufacturer's protocol. Cell viability was determined at 24, 48, 72 and 96 h by using the CellTiter 96 AQueous One Solution Cell Proliferation Assay kit (Promega, Madison, WI) according to the manufacturer's protocol. For colony formation assay, cells were seeded at low density (1000 cells/plate) and allowed to grow until visible colonies appeared. Then, cells were stained with Giemsa and colonies were counted. Cytoselect 24-well cell migration and invasion assay kit (Cell Biolabs, Inc) was used for migration and invasion assays according to manufacturer's protocol.

### Immunoblotting

Immunoblotting was performed as described previously [30]. The antibodies used were specific for *Akt* (4685; Cell Signaling), *p-Akt(Ser473)* (4060; Cell Signaling), PTEN (9552; Cell Signaling), P53 (2524; Cell Signaling), P73 (14620; Cell Signaling), p21 (2947; Cell Signaling) and *GAPDH* (sc-32233; Santa Cruz Biotechnology, Inc.). Blots were visualized using Western blotting luminal reagent (sc-2048; Santa Cruz Biotechnology, Inc.).

### Luciferase assays

The complimentary sites in 3′UTR of PTEN for miR-4534 and mutated sequences are given in Supplemental Table. The 3′-UTR region of PTEN containing target site sequences complementary to the seed sequence of miR-4534 were cloned downstream of the luciferase gene in the pMIR-REPORT luciferase vector (Ambion, Cambridge MA), and the resultant vectors named PTEN-3′UTR. Mutated 3′UTR sequences of PTEN complementary to miR-4534 were cloned in the same vector and the resultant vectors named PTEN-Mut 3′UTR. For reporter assays, cells were transiently transfected with wild-type or mutated reporter plasmid and miR-4534 or control-miR. Firefly luciferase activities were measured using the Dual Luciferase Assay (Promega, Madison, WI) 24 hr after transfection and the results were normalized with Renilla luciferase. Each reporter plasmid was transfected at least three times and each sample was assayed in triplicate.

### *In vivo* intratumoral delivery of anti4534

The antitumor effect of depletion of miR-4534 was determined by local administration of miR-4534 miRVANA miRNA inhibitor (antimiR) in established tumors in athymic nude mice and compared to antimiR-negative control mice group. Each mouse was injected sub-cutaneously with 2.0 ×10^6^ Du145 prostate cancer cells. Once palpable tumors developed, 6.25 μg of synthetic anti4534 or antimiR-negative control (control) complexed with 1.6 μl siPORT Amine transfection reagent (Ambion, Austin, TX) in 50 μl PBS was delivered eight times intratumorally at 3-day intervals. In total 8 mice received anti4534 and 8 mice received control miR. Tumor growth was followed for 21 days from the first injection. All animal care was in accordance with the institutional guidelines.

### Statistical analysis

Statistical analyses were performed with StatView for Windows (SAS Institute Inc. NC, USA), GraphPad-Pris- 5 and MedCalc. All quantified data represents average of at least triplicate samples and three experiments performed at different times or as indicated. Error bars represent standard deviation of mean or as indicated. All tests were performed two tailed and *p*-values < 0.05 were considered statistically significant. Receiver operating curves (ROC) were calculated to determine potential of miR-4534 to discriminate between malignant and non-malignant samples. Chi-square tests were performed to determine correlation between miR-4534 expression and clinicopathological characteristics.

## SUPPLEMENTARY MATERIALS TABLES


