# Supplementary material for: Oncogenic microRNA-4534 regulates PTEN pathway in prostate cancer
Source: Oncotarget. 2016 Sep 15;7(42):68371–84. doi: 10.18632/oncotarget.12031 (PMC5356562; doi:10.18632/oncotarget.12031)
Supplement: Supplementary file 1 [file oncotarget-07-68371-s001.pdf]

Oncogenic microRNA-4534 regulates PTEN pathway in prostate cancer

Supplementary Materials

Supplementary Table S1: Clinicopathologic characteristics of prostate cancer patients (*N* = 84)

Characteristics	Patient set†
**Age, Years**	**Number of patients (84)**
Mean	62
Median	68
Range	48–81
**T-stage**	
pT2	58
pT3-pT4	26
**Gleason Score**	
< 7	41
7	33
> 7	9
**PSA failure**	
Yes	34
**Pathological diagnosis**	
Adenocarcinoma	84

†miR-4534 expression determined from matched laser captured microdissected tissues.

Supplementary Table S2: Complimentary sequences in 3′UTRs of PTEN for miR-4534 and sequences of primers and probes

Complimentary sequences in 3′UTRs of PTEN for miR-4534 form miRBD

3′UCUGGGGAGGAGGUAG-G 5′miR-4534

| | | | | | | | | | | | |

5′TAACGACTT CTCCATCTC 3′ 911:PTEN 3UTR

3′UCUGGGGAGGAGGUAG-G 5′iR-4534

| | | | | | | | | | | | |

5′TATTGA AAT TAATCGATA 3′ 911:Mutant vector (Mutant PTEN 3UTR)

Underlined sequences in miR-4534mature sequence were changed for mutated 3′UTR (Mutant vector) sequences.

The primers for amplifying CpG islands are:

F1: 5′ GTGATTTATTTTTGTAAGTTTAGTATTTTGGGAG 3′(forward)

R1: 5′ ACAAATACRTACCACCATACCCAA 3′ (reverse)

The probes for methylation/unmethylation specific realtime PCR were synthesized by Applied Biosystems (Foster City, CA, USA), labeled with 6FAM reporter at 5′ end and with MGB quencher at 3′end. The sequences are:

M: 5′ CGAGGCGGGTAGATTATTTGAG 3′ (Methylation reaction)

U: 5′ TGAGGTGGGTAGATTATTTGAG (Unmethylation reaction)
